# Validation of use of billing codes for identifying telemedicine encounters in administrative data

**DOI:** 10.1186/s12913-019-4753-2

**Published:** 2019-12-03

**Authors:** Deepika Yeramosu, Florence Kwok, Jeremy M. Kahn, Kristin N. Ray

**Affiliations:** 1 Department of Pediatrics, University of Pittsburgh School of Medicine, Pittsburgh, PA USA; 2 Department of Epidemiology, University of Pittsburgh Graduate School of Public Health, Pittsburgh, PA USA; 3 Department of Critical Care Medicine, University of Pittsburgh School of Medicine, Pittsburgh, PA USA; 4 Department of Health Policy & Management, University of Pittsburgh Graduate School of Public Health, 3414 Fifth Avenue, Pittsburgh, PA 15213 USA

**Keywords:** Telemedicine, Validation, Accuracy, Claims

## Abstract

**Background:**

Telemedicine is the use of telecommunication technology to remotely provide healthcare services. Evaluation of telemedicine use often relies on administrative data, but the validity of identifying telemedicine encounters in administrative data is not known. The objective of this study was to assess the accuracy of billing codes for identifying telemedicine use.

**Methods:**

In this retrospective study of encounters within a large integrated health system from January 2016 to December 2017, we examined the accuracy of billing codes for identifying live-interactive and store-and-forward telemedicine encounters compared to manual chart review. To further examine external validity, we applied these codes and assessed patient and visit characteristics for identified live-interactive telemedicine encounters and store-and-forward telemedicine encounters in a second data set.

**Results:**

In manual review of 390 encounters, 75 encounters were live-interactive telemedicine and 158 were store-and-forward telemedicine. In weighted analysis, the presence of the GT modifier in the absence of the GQ modifier or CPT code 99444 yielded 100% sensitivity and 99.99% specificity for identification of live-interactive telemedicine encounters. The presence of either the GQ modifier or the CPT code 99444 had 100% sensitivity and 100% specificity for identification of store-and-forward telemedicine encounters. Applying these algorithms to a second data set (*n* = 5,917,555) identified telemedicine encounters with expected patient and visit characteristics.

**Conclusions:**

These findings provide support for use of CPT codes to perform telemedicine research in administrative data, aiding ongoing work to understand the role of non-face-to-face care in optimizing health care delivery.

## Background

Telemedicine encompasses the use of telecommunication technology to remotely provide healthcare services that support patient care [1]. Telemedicine can take many forms, with some models facilitating live interactive communication between patients and clinicians (“synchronous”) while other models record and send patient information, allowing clinicians to review and respond to this information at a later time (store-and-forward, or “asynchronous”). Both of these models have the potential to overcome geographic barriers and to enhance the timeliness of care. While these features certainly have appeal, potential concerns with telemedicine include the possibility of increased unnecessary visits due to the greater ease of access (contributing to increased health care costs), the potential for lower quality of care (particularly if clinical decisions are made with inadequate information), and the potential to perpetuate or exacerbate access disparities [2–4]. Thus, understanding the use, trends, and impact of telemedicine across multiple specialties and applications is vital to promoting telemedicine use that facilitates more effective, efficient, equitable, and patient-centered care delivery.

Administrative data are an important resource for understanding trends in telemedicine use and for assessing the effectiveness of telemedicine compared to other care delivery strategies. Administrative data analyses of telemedicine encounters rely on billing modifiers to identify telemedicine visits. Telemedicine billing modifiers were designated by the United States Centers for Medicare and Medicaid Services (CMS) to indicate encounters that used two specific types of telemedicine: live interactive telemedicine (GT) and store-and-forward telemedicine (GQ). Through identification of these codes, analyses of administrative data have contributed to our current understanding of telemedicine practices. For example, a recent analysis of a Minnesota All-Payers Claims Database elucidated a comprehensive picture of telemedicine use across multiple payer types and patient geographies [5]. Other studies used administrative data to study telemedicine for mental health services [6], substance use disorder treatments [7], primary care [8], and acute respiratory infections [9]. Prior analyses have also highlighted trends in telemedicine use among specific populations, such as rural communities [10] and children [11].

While the use of billing codes within administrative data to identify telemedicine encounters are widely used, we are not aware of any validation studies comparing these codes to a gold standard of chart review. Understanding the sensitivity and specificity of these codes for identification of telemedicine in general, as well as for synchronous versus asynchronous telemedicine specifically, will allow for clearer understanding of the strengths and limitation of this approach for identification of telemedicine encounters. Thus, in this study, we assessed the validity of using billing modifiers to identify telemedicine encounters within a large integrated health system.

## Methods

### Study design and data sources

Our study had two parts. First, we examined the accuracy of billing codes in identifying telemedicine encounters, the goal of which was to determine the criterion validity of use of these billing codes (i.e., to what degree do these codes reflect the occurrence of a telemedicine encounter?). To accomplish this, we performed manual chart review and compared billing codes to chart review findings, allowing determination of sensitivity and specificity of specific combinations of billing codes for telemedicine encounters and refinement of billing code algorithms for identifying telemedicine encounters.

Second, we examined the frequency with which telemedicine encounters were identified using billing code algorithms, overall and by encounter characteristics, to provide external validity for use of these billing codes in a separate data set (i.e., After applying the validated algorithms to another data set, to what degree do identified telemedicine encounters reflect expected characteristics of telemedicine encounters?). To accomplish this, we applied the billing code algorithms with best performance during the prior step, and compared the patient and visit characteristics of identified telemedicine encounters.

For both parts of this analysis, we used data from a 40-hospital, 4900-physician integrated health system spanning Western Pennsylvania. Multiple electronic health records are used across inpatient and outpatient services within this system, and our analysis was not limited to a specific electronic health record or a specific telemedicine platform. Data were obtained from University of Pittsburgh Medical Center (UPMC) clinical data obtained through the University of Pittsburgh Health Record Research Request (R3) Service. For the first part of the study, we sampled encounters from a data set from January 1 – June 30, 2016. For the second part of the study, we used a complete encounter data set from July 1, 2016 – December 31, 2017. Analyses were conducted in StataSE 14 (StataCorp), and this project was approved by the University of Pittsburgh Institutional Review Board.

### Candidate components of telemedicine identification algorithms

Prior to analysis, we identified multiple potential components of algorithms for identification of telemedicine, based on review of prior approaches [5, 8, 10] and on insurer bulletins [12]. Specifically, we focused on two specific modifiers (GT and GQ), which were developed for indication of live interactive telemedicine and store-and-forward telemedicine, respectively. Additionally, we investigated additional CPT codes indicating telemedicine and online services (e.g., 99,444, G0425-G0427) delivered by a physician, nurse practitioner, or physician assistant services. We did not investigate CPT codes designed for use by allied health professionals (e.g., G0270) to allow more focused study and because of the potential for such services to be documented outside of systems available for manual review.

### Chart review

For the chart review, we began with UPMC clinical data for all encounters between January 1–June 30, 2016. Encounters were identified using common procedural terminologies (CPT) codes indicating psychiatry visits (90,791, 90,792), psychotherapy visits (90832–90847), outpatient visit (99201–99205, 99211–99215), inpatient hospitalization (99217–99239), outpatient consultation (99241–99245), inpatient consultation (99251–99255), emergency department (99281–99285), telephone encounter (99441–99443) online visit (99444), and telehealth consultation (G0406-G0408; G0425-G0427; G0508-G0509). Wellness or preventive health visits were not included, as those visits were presumed to be less likely to incorporate telemedicine. We did not exclude visit encounters based on patient age.

From this set of encounters, a random stratified sample of 390 encounters was selected for chart review. To ensure adequate representation of different combinations of billing codes in the sample, we randomly sampled approximately 130 encounters with the GT modifier, 130 with the GQ modifier, and 130 with neither modifier, with this sample size set to provide estimates with a margin of error at or below 5%. Within each modifier group (GT, GQ, and neither), we sampled an equal number of inpatient and outpatient encounters (if available) to ensure sampling of encounters across potential sources of variability in coding accuracy (Table 1). The GQ modifier was only associated with outpatient billing codes, however, and therefore all 130 encounters with the GQ modifier were sampled from outpatient billing code encounters.

**Table 1:** Encounter Review Sample Strata and Sample Weights

Billing Code Criteria	Number of Encounters Available	Proportion of Available Encounters	Number of Encounters Selected	Proportion of Selected Encounters	Sample Weight^a^
Inpatient encounter, no modifier code	796,481	0.379	63	0.1615	2.349146
Outpatient encounter, no modifier code	1,299,521	0.619	67	0.1718	3.603393
Inpatient encounter, GT modifier	552	0.000263	65	0.1667	0.001578
Outpatient encounter, GT modifier	2076	0.000989	62	0.1590	0.006222
Inpatient encounter, GQ modifier	0	0	0	0	0
Outpatient encounter, GQ modifier	258	0.000123	132	0.3385	0.000363

^a^Sample weight = (Percent of encounters available) / (Percent of encounters selected)

Manual review of two electronic medical record systems were then conducted by trained reviewers using a structured instrument to assess and record whether the selected encounter was completed through telemedicine, whether telemedicine used was synchronous or asynchronous, as well as additional features of telemedicine encounters, when identified. We determined whether the visit was telemedicine and what type of telemedicine encounter first by reviewing the full text of the clinical documentation, second by searching specifically for common keywords and phrases in the encounters (“telemedicine”, “audio”, “visual”, “electronic”), and third by specifically reviewing the exam documentation for indication of in-person versus remote evaluation. Any indication of telemedicine through these three steps was considered sufficient to categorize that visit as a telemedicine encounter.

Among visits determined to be telemedicine (either live interactive or store-and-forward), we also extracted additional elements to examine the specific contexts of telemedicine use. Specifically, to understand the context of the telemedicine encounter relative to in-person care, we extracted whether any prior or follow-up visits occurred with the same specialty in the year prior or the year subsequent to the telemedicine encounter.

To achieve high reliability in this manual review of encounters, we first had two reviewers (DY, KNR) review 6 training encounters outside of the sample, after which discrepancies were discussed encounter by encounter. Then the first 20 encounters in the sample were independently reviewed by these 2 reviewers with discrepancies discussed, after which the next 40 encounters were again reviewed independently by 2 reviewers with discrepancies discussed. The Cohen’s kappa statistic for the first set of 20 was 0.92 and for the second set of 40 was 1.00, indicating a high level of agreement between reviewer. The remaining encounters were reviewed by a single reviewer (DY). Encounters were reviewed in a random order, and the reviewer was blinded to the billing codes for each encounter. Sampled encounters with no available clinical documentation were replaced with another randomly-selected encounter from the same sampling strata, with 2 additional GQ encounters, no additional GT encounters, and 22 additional encounters with neither modifier sampled. Data were entered into REDCap electronic data capture tools hosted at the University of Pittsburgh.

### Chart review analysis

Results from manual review of encounters were weighted to account for proportion of each strata in the available encounters relative to the sampled encounters (Table 1). Applying survey functions to account for these weights, we calculated sensitivity and specificity of these billing codes for identification of telemedicine visits, including 95% confidence intervals (CIs). We determined these values for multiple combinations of telemedicine types (live interactive telemedicine, store-and-forward, or either) and multiple permutations of billing codes (GT or GQ as well as specific sets of CPT codes; Table 2), allowing manual identification of preferable algorithms for identifying telemedicine encounters. We also determined the positive predictive value (PPV) and negative predictive value (NPV) for each combination of billing codes. After selecting the preferred algorithm for identification of each type of telemedicine visit in the overall data, we further determined the sensitivity, specificity, PPV, and NPV for these algorithms within specific subsets of visits identified by CPT codes (e.g., inpatient, outpatient, etc.). Finally, we used descriptive statistics to compare encounter review findings for live-interactive versus store-and-forward telemedicine in terms of prior and follow-up visits within the same department.

**Table 2:** Diagnostic Agreement Between Different Billing Code Algorithms and Encounter Review Gold Standard

Encounter Review Finding	Claims Algorithm	Sensitivity	Specificity	PPV	NPV
Any Telemedicine	GT or GQ present	100% (−-)	99.99% (99.98–99.99%)	90.8% (86.1–94.0%)	100% (−-)
	GT or GQ or 99444 present	100% (−-)	99.99% (99.98–99.99%)	90.8% (86.1–94.0%)	100% (−-)
Live Interactive Telemedicine	GT present	100% (−-)	99.93% (99.92–99.95%)	51.4% (42.0–60.7%)	100% (−-)
	GT present without GQ or 99444	100% (−-)	99.99% (99.98–99.99%)	84.8% (76.9–90.3%)	100% (−-)
Store and Forward Telemedicine	GQ present	36.5% (25.3–49.3%)	100% (100–100%)	99.1% (96.3–99.8%)	99.96% (99.95–99.98)
	GQ or 99444	100% (−-)	100% (100–100%)	99.7% (98.6–99.9%)	100% (−-)

Note: Abbreviations: *PPV* positive predictive value; *NPV* negative predictive value

### External validation data set

The second set of administrative data was obtained for the 18 months after the initial data set (July 1, 2016-December 31, 2017). Encounters were again identified for inclusion based on the same CPT codes as the prior data set. Encounters were not excluded based on any patient age. Variables in this second data set included billing codes, billing modifiers, patient age, race/ethnicity, insurer, ZIP code, visit date, provider and specialty department. Insurer type was categorized as commercial, public (Medicaid, Medicare), and self-pay/missing. Straight line distance from patient ZIP code centroid to the primary tertiary medical center location was determined using Stata calculations. County rural/urban status was determined using USDA rural-urban continuum codes [13]. Visit dates were categorized into quarters. Clinician specialty were summarized into the following groups: primary care, emergency medicine, dermatology, pediatric specialties, non-pediatric medical specialties, non-pediatric surgical specialties, psychiatry, and other.

In this second data set, we determined whether a telemedicine visit occurred by applying the most sensitive and specific algorithms for live interactive telemedicine and store-and-forward telemedicine determined through the prior analysis. Specifically, live interactive telemedicine was defined as the presence of the GT modifier in the absence of the GQ modifier and the absence of the 99444 CPT code. Store-and-forward telemedicine was defined as the presence of the GQ modifier or the 99444 CPT code, regardless of the presence of the GT modifier. Telemedicine of either type was defined as the presence of the GT modifier, the GQ modifier, or the 99444 CPT code.

### External validation analysis

Using chi-square tests, we compared visit characteristics for non-telemedicine visits, live interactive telemedicine visits, and store-and-forward visits to determine external validity in this separate data set. We also assessed the unique number of providers delivering care via each type of visit.

Based on prior studies [6] and insurer policies [14], we hypothesized that live interactive telemedicine would be more prevalent among individuals residing greater distances from the main tertiary medical center and residing in rural counties. We hypothesized that store-and-forward telemedicine would be more prevalent among younger adults than older adults, because younger age patients have been reported in prior evaluations of store-and-forward dermatology encounters [15]. We hypothesized that both types of telemedicine would be more prevalent in more recent quarters given reports of increasing telemedicine use [8, 11].

## Results

### Chart review

The first data set included 2,096,002 encounters without GT or GQ modifiers, 2,628 with the GT modifier, and 258 with the GQ modifier. Among these, a sample of 390 were reviewed. Through manual encounter review of these 390 encounters, we identified 75 live interactive telemedicine visits, 158 store-and-forward telemedicine visits, and 157 visits that were not telemedicine. Of note, 5 of the encounters sampled based on the presence of the GT modifier also contained the GQ modifier (4%), and 108 of the encounters sampled based on the presence of the GQ modifier also contained the GT modifier (82%).

After adjusting for prevalence of each sampling strata through survey weights, the presence of either the GT and/or GQ modifier had 100% sensitivity and 99.99% specificity (95% CI 99.98–99.99%) for broadly identifying telemedicine encounters (Table 2), including either store-and-forward or live interactive telemedicine visits. In this sample, the positive predictive value (PPV) was 90.8% (95% CI 86.0–94.0%) and the negative predictive value (NPV) was 100%.

For identification of live interactive telemedicine visits, the presence of the GT modifier had 100% sensitivity and 99.93% specificity (95% CI 99.92–99.95%). Identifying live-interactive telemedicine with the presence of GT modifier after excluding cases that also had the GQ modifier or the CPT code 99444 improved the specificity to 99.99% (95% CI 99.98–99.99%) while maintaining 100% sensitivity, making this the preferred algorithm for live interactive telemedicine identification. Because telehealth codes (G0406-G0408; G0425-G0427; G0508-G0509) consistently co-occurred with the GT modifier in this sample, algorithm sensitivity/specificity neither improved nor worsened with the addition of these codes into identification algorithms.

For identification of store-and-forward telemedicine visits, the presence of the GQ modifier had 36.36% sensitivity (95% CI 25.2–49.3) and 100% specificity. Using the presence of either the GQ modifier or the CPT code 99444 had 100% sensitivity and 100% specificity for identification of store-and-forward telemedicine visits, making this the preferred algorithm for store-and-forward identification.

Among only outpatient visits, the presence of either the GT and/or GQ modifier had 100% sensitivity and 99.99% specificity (95% CI 99.98–100%), with PPV of 97.0 (95% CI 89.3–99.2) and NPV of 100% (Table 3). When examining only inpatient visits, the presence of either the GT and/or GQ modifier had 100% sensitivity and 99.98% specificity (95% CI 99.97–99.98%), with 64.6% PPV (95% CI 52.1–75.4%) and 100% NPV.

**Table 3:** Diagnostic Agreement Between Optimized Billing Code Algorithms and Encounter Review Gold Standard for Specific Encounter Types

Encounter setting	Sensitivity	Specificity	PPV	NPV
Any telemedicine visit (using GT, GQ, or 99444)				
All inpatient & outpatient encounters	100% (−-)	99.99% (99.98–99.99%)	90.8% (86.1–94.0%)	100% (−-)
All outpatient encounters (visits, consultations, and online)	100% (−-)	99.99% 99.98–100%)	97.0% (89.3–99.2%)	100% (−-)
- Problem-based outpatient visit	100% (−-)	99.99% (99.98–100%)	88.9% (63.3–97.4%)	100% (−-)
All inpatient encounters (encounters, consultations, emergency)	100% (−-)	99.98% (99.97–99.98%)	64.6% (52.1–75.4%)	100% (−-)
- Inpatient encounter	100% (−-)	99.98% (99.97–99.99%)	55.2% (36.6–72.4%)	100% (−-)
- Inpatient consultation	100% (−-)	99.9% (99.7–99.97%)	66.7% (26.8–91.6%)	100% (−-)
Live interactive telemedicine (using GT in absence of GQ or 99444)				
All inpatient & outpatient encounters	100% (−-)	99.99% (99.98–99.99%)	84.8% (76.9–90.3%)	100% (−-)
All outpatient encounters (visits, consultations, and online)	100% (−-)	99.99% (99.98–100%)	94.3% (79.5–98.6%)	100% (−-)
- Problem-based outpatient visit	100% (−-)	99.99% (99.98–100%)	88.9% (63.3–97.4%)	100% (−-)
All inpatient encounters (encounters, consultations, emergency)	100% (−-)	99.98% (99.97–99.98%)	64.6% (52.1–75.4%)	100% (−-)
- Inpatient encounter	100% (−-)	99.98% (99.97–99.99%)	55.2% (36.6–72.4%)	100% (−-)
- Inpatient consultation	100% (−-)	99.9% (99.7–99.97%)	66.7% (26.8–91.6%)	100% (−-)
Store and Forward Telemedicine (using GQ or 99444)				
All inpatient & outpatient encounters	100% (−-)	100% (100–100%)	99.7% (98.6–99.9%)	100% (−-)
All outpatient encounters (visits, consultations, and online)	100% (−-)	100% (100–100%)	99.7% (98.6–99.9%)	100% (−-)

Note: Abbreviations: *PPV* positive predictive value; *NPV* negative predictive value

Using encounter review data, we also compared overall clinical context of live-interactive versus store-and-forward telemedicine encounters. In general, live-interactive telemedicine visits more often occurred within the context of ongoing clinical relationships, while store-and-forward visits more often occurred as isolated encounters. Specifically, from unweighted encounter review results, the percentage of encounters in which patients had a prior in-person visit in the same department was 23% among live interactive telemedicine encounters, compared to 8% of store-and-forward telemedicine encounters (*p* = 0.002). Similarly, the percentage of encounters in which patients had a subsequent follow-up visit in the same department (in-person and/or telemedicine) was 57% among live interactive telemedicine encounters and 33% of store-and-forward encounters (*p* < 0.001).

### External validation

We then applied the preferred telemedicine identification algorithms to the second dataset to examine external validity of this algorithm outside of the original chart review data set. The second data set (July 1, 2016-December 31, 2017) included 5,917,555 encounters with over 6000 clinicians. Among these encounters, 888,365 (15%) were by ≤17-year-old patients and 2,138,653 (36%) were by > 65-year-old patients (Table 4). Similar proportions of encounters were covered by commercial (47%) and public insurance (49%).

**Table 4:** Characteristics of Identified Telemedicine Encounters in Validation Data Set

	Not Telemedicine	Live Interactive Telemedicine	Store and Forward Telemedicine	
Administrative Code Algorithm	No GT or GQ Modifier AND not CPT code 99444	GT modifier in absence of both GQ and CPT code 99444	GQ modifier or CPT code 99444	
N	5,909,464	3087	5004	p
Patient Age (years)				< 0.001
0–17	888,045 (15)	57 (2)	263 (5)	
18–24	314,227 (5)	109 (4)	339 (7)	
25–44	968,258 (16)	753 (24)	2769 (55)	
45–64	1,601,736 (27)	875 (28)	1470 (29)	
> 65	2,137,198 (36)	1293 (42)	163 (3)	
Patient Race				< 0.001
Black	666,111 (11)	132 (4)	198 (4)	
White	4,998,279 (85)	2910 (94)	4095 (82)	
Other	70,542 (1)	15 (1)	92 (2)	
Missing	174,532 (3)	30 (1)	619 (12)	
Patient Insurance				< 0.001
Commercial	2,757,532 (47)	1044 (34)	4159 (83)	
Public	2,899,994 (49)	1963 (64)	270 (5)	
Self-Pay or Missing	251,938 (4)	80 (3)	575 (12)	
Distance to tertiary care center (miles)				< 0.001
0–9	1,993,372 (34)	141 (5)	2096 (42)	
10–19	989,090 (17)	90 (3)	1119 (22)	
20–29	509,346 (9)	39 (1)	534 (11)	
30–59	803,624 (14)	526 (17)	506 (10)	
60–89	841,962 (14)	2006 (65)	339 (7)	
≥90	709,626 (12)	230 (7)	338 (7)	
Missing	62,444 (1)	55 (2)	72 (1)	
Residential County Rural/Urban Status				< 0.001
Large metropolitan	3,912,918 (66)	342 (11)	4050 (81)	
Small Metropolitan	1,169,892 (20)	713 (23)	472 (9)	
Non-Metropolitan	826,654 (14)	2032 (66)	482 (10)	
Encounter Date				< 0.001
2016 Quarter 3 (July – Sept)	1,004,107 (17)	467 (15)	699 (14)	
2016 Quarter 4 (Oct – Dec)	991,931 (17)	430 (14)	635 (13)	
2017 Quarter 1 (Jan – Mar)	999,649 (17)	491 (16)	935 (19)	
2017 Quarter 2 (Apr – June)	991,547 (17)	572 (19)	768 (15)	
2017 Quarter 3 (July – Sept)	1,002,313 (17)	495 (16)	838 (17)	
2017 Quarter 4 (Oct – Dec)	919,917 (16)	632 (20)	1129 (23)	
Encounter Provider Specialty				< 0.001
Primary Care	1,956,710 (33)	48 (2)	46 (1)	
Emergency Medicine	998,362 (17)	0 (0)	4240 (85)	
Dermatology	91,973 (2)	0 (0)	717 (14)	
Medical Specialty	1,442,710 (24)	2247 (73)	1 (0)	
Surgical Specialty	879,567 (15)	580 (19)	0 (0)	
Pediatric Specialty	96,208 (2)	67 (2)	0 (0)	
Psychiatry	205,831 (4)	129 (4)	0 (0)	
Other	238,103 (4)	16 (1)	0 (0)	
Unique Providers, number	6522	103	76	NA

Note: Abbreviations: *CPT* current procedural terminology

Visits identified as live-interactive telemedicine were associated with expected encounter characteristics. Specifically, live-interactive telemedicine visits were more likely to be with patients living farther from the tertiary care center (60–90 miles: 65% of live interactive telemedicine visits versus 14% of in-person visits; Table 4). Live interactive telemedicine visits were also more commonly from non-metropolitan communities (66% of live interactive telemedicine encounters versus 14% of in-person visits). Additionally, a greater percentage of live interactive telemedicine visits occurred in the most recent quarter (20% in the fourth quarter of 2017, increased from 15.2% in the third quarter of 2016). These findings were consistent with our expectations for live-interactive telemedicine visits. Additionally, live-interactive telemedicine encounters were predominantly associated with consultation CPT codes (inpatient, outpatient, and telehealth) and with age > 65 years old (42%).

Visits identified as store-and-forward telemedicine encounters were also associated with expected encounter characteristics. Specifically, these encounters were most likely to occur among young adults (25–44-year-old: 55% of store-and-forward encounters versus 16% of in-person encounters). Encounters identified as store-and-forward were also predominantly in more recent quarters (fourth quarter of 2017: 23% of all store-and-forward encounters, increased from 14% in the third quarter of 2016). These findings further support the validity of the identification algorithm in this category as well. Additionally, we found that store-and-forward telemedicine encounters were more likely to occur with patients in close proximity to the tertiary care center.

## Discussion

With increasing interest in and use of telemedicine, health services researchers require accurate means to identify telemedicine encounters in order to identify trends in use, clarify disparities in use, and determine impact on health outcomes. In this analysis, we examine the validity of using specific modifiers and billing codes to identify any telemedicine encounter and to identify specific subtypes of telemedicine encounters (live-interactive and store-and-forward). Using GT, GQ, and the 99444 modifiers together to identify any telemedicine encounter had 100% sensitivity and 99.99% specificity for telemedicine encounters. Our analysis provides strong support for use of these codes in telemedicine research, essential for ongoing work to understand role of non-face-to-face care in optimizing health care delivery.

Through this analysis, we examined criterion validity for identification of telemedicine encounters with billing codes, and we refined algorithms to achieve high sensitivity and specificity. We subsequently further assessed external validity by examining the degree to which identified telemedicine encounters were associated with expected patient and visit characteristics in a second data set. Results in this second phase of our analysis were consistent with hypothesized findings, providing evidence of external validity. Specifically, we anticipated increased use of live-interactive and of store-and-forward telemedicine over time, which we observed in these data. Also consistent with our expectations, live interactive telemedicine encounters were more commonly observed among patients who lived in more rural communities and at greater distance from the tertiary care center. These findings are consistent with prior literature [5, 6] and consistent with Medicaid and Medicare policy during the study period [14]. Specifically, Medicare telemedicine reimbursement was limited to health professional shortage areas during the study period, and Pennsylvania Medicaid policy in place during the study period suggests that providers should consider travel time greater than 60 min in rural areas or greater than 30 min in an urban area when considering telemedicine use [14]. Also consistent with prior studies and expected findings, we observed higher use of store-and-forward telemedicine among younger adults compared to older adults. Altogether, these findings provide additional support for the validity of use of billing claims to identify telemedicine and to differentiate between live-interactive and store-and-forward telemedicine.

We also observed additional associations between patient characteristics and use of each subtypes of telemedicine beyond those we hypothesized. These additional findings add further to our understanding of the use of live-interactive telemedicine versus store-and-forward telemedicine. For example, live interactive telemedicine encounters were more common among adults over 65 years of age and publicly insured individuals. The consistency in federal regulations governing use of live interactive telemedicine among Medicare recipients – in contrast to the complexity of policies and practices among commercial insurers and Medicaid managed care organizations – may contribute to this finding. In contrast, store-and-forward telemedicine use appeared to be primarily among commercially insured individuals. Additionally, the patients receiving telemedicine encounters were disproportionately white (94% of live interactive telemedicine encounters; 93% of store-and-forward telemedicine encounters with non-missing race), raising concerns about whether use of telemedicine is enhancing access uniformly versus perpetuating disparities.

In examining the clinical context of telemedicine visits, it appears that specialties primarily offer one of the two models of telemedicine, rather than both. For example, dermatology and emergency medicine department clinicians provided a large number of store-and-forward encounters and no live-interactive telemedicine encounters. In contrast, medical specialties and surgical specialties provided the bulk of live-interactive telemedicine encounters and rare store-and-forward encounters. This may be appropriate adoption of the technology that best fits a field’s clinical care, but may also reflect the difficulties behind starting telemedicine service lines, requiring departments to focus on one technology and workflow at a time. From our manual review of encounters, we also observed different longitudinal contexts of each telemedicine type, with live interactive telemedicine associated more often than store-and-forward telemedicine with prior in-person visits and with subsequent follow-up visits. These additional findings help to characterize the contexts of live interactive and store-and-forward telemedicine use within a large health care system, adding to the main findings which validate use of claims for identification of telemedicine encounters.

One limitation of our analysis is that we examined encounters within one health system. However, the health system includes over 40 hospitals and thousands of providers across a large geographic area, and we used separate data from a more recent time period for the second phase of our analysis. Additionally, telemedicine encounters remain a small percentage of overall care in this system, such that there is the potential for billing and coding to be relatively idiosyncratic – however, we did identify 103 unique providers contributing to live interactive telemedicine claims and 76 unique providers contributing to store-and-forward claims. An additional limitation is that telemedicine billing continues to evolve, with Medicare adding unique telemedicine place of service codes in 2018 and proposing additional telemedicine-specific encounter codes in 2019 [12]. While future work may need to update this analysis, our analysis remains an important step in validating the most common billing codes in current analyses. Finally, we note that because positive and negative predictive value are influenced by prevalence, the PPVs and NPVs will not be applicable to health systems with telemedicine use that differs significantly from the prevalence observed in our system.

## Conclusion

We identified algorithms with high sensitivity and specificity for identification of telemedicine encounters overall and for identification specifically of live-interactive telemedicine and store-and-forward telemedicine encounters, and we provide addition evidence of validity through assessment of the association of each type of telemedicine encounter with expected patient and visit characteristics. These findings provide strong methodological support for use of CPT codes in telemedicine research, essential for ongoing work to understand the role of non-face-to-face care in optimizing health care delivery.

## Data Availability

The datasets analyzed during the current study are available from the corresponding author on reasonable request and with approval of the University of Pittsburgh Institutional Review Board.

## Abbreviations

CI: Confidence interval
CPT: Current Procedural Terminology
NPV: Negative predictive value
PPV: Positive predictive value
UPMC: University of Pittsburgh Medical Center
